# Niños Sanos, Familia Sana: Mexican immigrant study protocol for a multifaceted CBPR intervention to combat childhood obesity in two rural California towns

**DOI:** 10.1186/1471-2458-13-1033

**Published:** 2013-10-31

**Authors:** Adela de la Torre, Banafsheh Sadeghi, Richard D Green, Lucia L Kaiser, Yvette G Flores, Carlos F Jackson, Ulfat Shaikh, Linda Whent, Sara E Schaefer

**Affiliations:** 1 Department of Chicana/o Studies, University of California, Davis, One Shields Avenue, Davis, CA 95616, USA; 2 Department of Internal Medicine, School of Medicine, University of California, Davis, 4150 V Street, PSSB Suite 2400, Sacramento, CA 95817, USA; 3 Department of Agricultural & Resource Economics, University of California, Davis, One Shields Avenue, Davis, CA 95616, USA; 4 Department of Nutrition, University of California, Davis, One Shields Avenue, Davis, CA 95616, USA; 5 Department of Pediatrics, School of Medicine, University of California, Davis, 2516 Stockton Blvd, Suite 340, Sacramento, CA 95817, USA; 6 Foods for Health Institute, 2141 RMI North, University of California, Davis, CA 95616, USA

**Keywords:** Multi-faceted intervention, Community-based participatory research, Latino health, Childhood obesity prevention

## Abstract

**Background:**

Overweight and obese children are likely to develop serious health problems. Among children in the U.S., Latino children are affected disproportionally by the obesity epidemic. Niños Sanos, Familia Sana (Healthy Children, Healthy Family) is a five-year, multi-faceted intervention study to decrease the rate of BMI growth in Mexican origin children in California’s Central Valley. This paper describes the methodology applied to develop and launch the study.

**Methods/Design:**

Investigators use a community-based participatory research approach to develop a quasi-experimental intervention consisting of four main components including nutrition, physical activity, economic and art-community engagement. Each component’s definition, method of delivery, data collection and evaluation are described. Strategies to maintain engagement of the comparison community are reported as well.

**Discussion:**

We present a study methodology for an obesity prevention intervention in communities with unique environmental conditions due to rural and isolated location, limited infrastructure capacity and limited resources. This combined with numerous cultural considerations and an unstable population with limited exposure to researcher expectations necessitates reassessment and adaptation of recruitment strategies, intervention delivery and data collection methods. Trial registration # NCT01900613.

**Trial registration:**

NCT01900613.

## Background

Despite recent evidence suggesting slight declines in low-income, child obesity rates in the United States, these trends are not shared equally across subpopulations and high rates of childhood obesity are still prevalent across the United States [1]. Based on the most recent National Health and Nutrition Examination Survey (NHANES), approximately one in six children and adolescents in the U.S., aged 2–19 y, are obese [2]. The prevalence of obesity disproportionately affects certain U.S. ethnic groups, including Latino-Americans. Among children and adolescent populations, obesity affects 21.2% of Latinos compared to 14% white non-Latinos [2]. Such differences suggest that more information is needed to assess childhood obesity risk and preventive factors in order to create culturally appropriate and sustainable behavioral interventions for high-risk populations.

Scant data exist on the efficacy of school- and community-based interventions targeting obesity prevention and healthy lifestyles for Latino children, resulting in a critical gap in understanding how multifaceted interventions impact behavioral change in these communities [3]. While nutrition education interventions targeting Latino parents of young children have reported improvement in Body Mass Index (BMI), it is unclear whether these approaches can be scaled-up and achieve sustainable results [4,5]. *Aventuras para Niños*, a community-based study conducted in San Diego, did not find significant improvement in BMI z-scores over the three-year follow-up study [6]. Although the San Diego study included family- and community-level components, additional economic incentives, for example similar to that used in the United States Department of Agriculture’s (USDA) Women, Infant and Children (WIC) program, may be needed to overcome financial barriers to achieving a healthy diet and lifestyle.

In attempt to address the dearth of literature in the field of childhood obesity prevention for the growing Latino community and data suggesting that underlying community factors may impact these rates, we develop *Niños Sanos, Familia Sana* (NSFS), a five-year multifaceted intervention study targeting California’s Mexican-origin communities. The research goals include (a) identifying individual and environmental factors that influence food consumption patterns; and (b) generating new knowledge about community- and school- based interventions to reduce the rate of growth of BMI within Mexican-origin children. The behavioral intervention includes curriculum-based interventions in the community (preschool) and schools (K-2). A companion parent education and market-based (fruit and vegetable vouchers) intervention addresses the reality that parents make food choices for young children but may not have the resources to make healthy choices.

This study includes a novel interdisciplinary theoretical model to explain the stages of, and influences on, behavioral change within the targeted community. To design the intervention, we integrate social cognitive theory (SCT) and an economic behavioral approach within the Health Belief Model (HBM) [7,8]. These models and theories enable us to focus on behavioral changes through multiple levels of influence, such as the individual, interpersonal and community levels [9]. The introduction of the economic behavioral assumptions within the context of the SCT and HBM is novel. Based on the interdisciplinary theoretical approach, we shifted away from a single intervention to a multifaceted intervention approach to explore the capacity for behavioral changes. To our knowledge this intervention approach is unique, as school-based and other community-based studies targeting the Mexican-origin population to date have focused on single and/or short-term interventions to create behavioral changes. Recent literature suggests that successful interventions must take place in a community setting that calls upon the involvement and “buy-in” of all stakeholders within that setting [10]. This approach addresses the problem of Mexican-origin childhood overweight/obesity within the context of a local community and allows us to partner effectively with the school and community, facilitating better awareness of the problem among all stakeholders. It also fosters participants’ abilities to work with us in tailoring the proposed interventions to meet their cultural, social and economic needs. Since this interdisciplinary model employs a community-based participatory research (CBPR) approach, input is required at all stages of school and community engagement, as well as in the development and implementation of the proposed interventions [11-15]. Thus our intervention is based in the local schools, community centers, food markets and health clinics.

A major goal of *Niños Sanos, Familia Sana* is to reduce the rate of growth of childhood obesity among Mexican-origin children between the ages of three and eight by improving the economic capacity of low-income, Mexican-origin families, enhancing the nutritional skills of families, supporting the capacity of schools to more effectively provide Physical Education (PE) opportunities for children in preschool and K-2 settings, and providing community art programs that allow for nutrition and positive behavioral health messaging. Biannual anthropometric data, annual physical activity data and annual household survey data are collected of our sample population, which allow us to determine the impact of behavioral and community intervention factors on rate of growth of childhood obesity within our study population. These data are compared to our comparison community that receives a non-nutrition community-based intervention.

## Methods

### Project design

**Figure 1:**
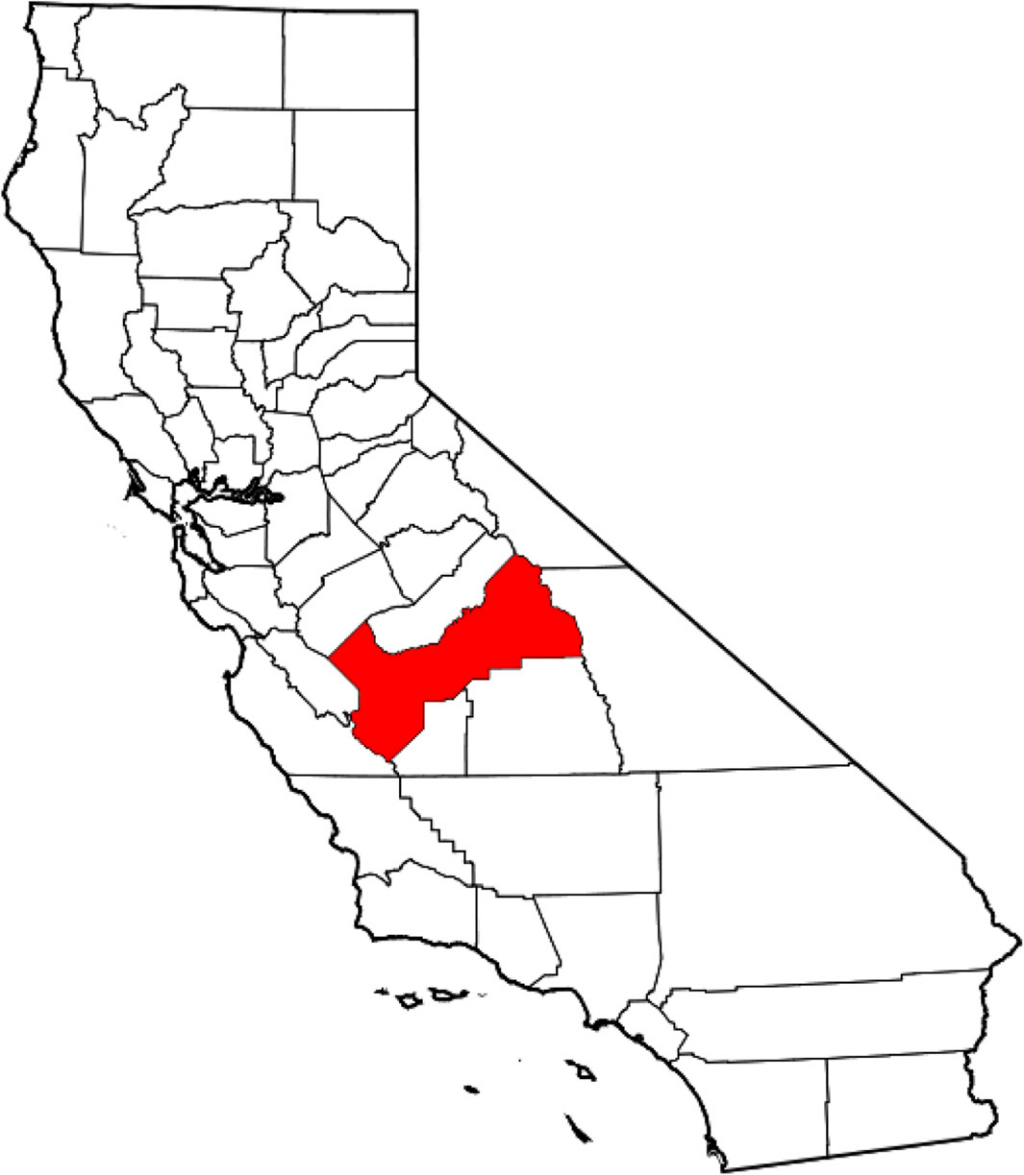
Map of California highlighting Fresno county.

The quasi-experimental design of this five-year intervention study allows us to match two communities in California’s Central Valley with similar demographic characteristics. Both towns are over 80% Mexican-origin and the targeted school communities are predominantly Mexican origin. The primary employment base is agricultural, and the community members have similarly low levels of educational attainment. Two additional essential criteria for the project are local community leadership involvement with the University of California, Davis (UCD) research team and no active comprehensive nutrition intervention present in either town. Based on these criteria and after completion of detailed environmental scans to ensure close community matching, the towns of San Joaquin and Firebaugh located in Fresno County, California (Figure 1) are selected, including their respective school districts Golden Plains and Firebaugh-Las Deltas. Prior to initiation of this research study, the UCD research team cultivated a relationship of mutual trust and respect in each town over the course of a year through a series of targeted meetings with community leaders including city managers, local elected officials, school superintendents and boards, teachers, religious leaders, parents and local health care professionals. The research team presented the proposed research at town hall presentations in each site to introduce and explore the project concept and receive feedback from community participants. Once the key community stakeholders agreed on project participation, the intervention and comparison community were randomly selected. Throughout the process of community engagement, a major goal of the research team is solidification of community understanding of the project by key actors, and establishment of clear communication and agreements among community leaders. In order to facilitate this process of community participation, a Community Advisory Council (CAC) of stakeholders from each community was recruited for the project in consultation with the university research team. Both councils include the following representatives: city manager, school superintendents, teachers, principals, school nurses, food service managers, representatives of the local health facility, religious leaders, community health outreach workers (*promotores*), and representation from a major local supermarket. Local *promotores* are employed to assist with broad based outreach and recruitment for all town hall and community events. Throughout the duration of the project, the CAC meets quarterly with the research team and members are invited to participate in all community events.

The five-year study design allows for the first year to be used as a planning and start up year, which is critical for piloting all interventions and study instruments prior to implementation. In addition, this baseline year is critical for recruitment of households with eligible children. In both instances, input from the CACs is critical to provide recommendations to enhance recruitment strategies and participation of eligible applicants into the study.

### Subject eligibility and recruitment

*Promotores* are hired in both communities to facilitate recruitment of families and trained to conduct recruitment events and go house to house to recruit hard-to-reach families. Subject eligibility for this project is determined a priori by the USDA’s National Institute of Food and Agriculture (NIFA) guidelines stated in the request for proposal. Eligible applicants for the study include children who fall within the ages of 3 to 8 years of age during the intervention period years. During the first year of the study, families with children between the ages of 2–5 are recruited as these individuals are within the guideline requirements (Table 1).

**Table 1:** Inclusion and exclusion criteria for family participation in NSFS study

**Criteria**	**Description**
Inclusion criteria:	• Have at least one child residing at home in the required age/grade level range. Children must be born between December 3, 2004 and October 1, 2009, or must be in 2nd grade or lower during the 2012–2013 school year and
	• Residence in the Firebaugh Las Deltas Unified School District (FLDUSD) or the Golden Plains School Unified District (GPUSD)
Exclusion criteria:	• Family/child not interested in participating or
	• Moved out of school district or
	• Child no longer living at home or
	• Not enough time to participate in required activities

To facilitate recruitment, all children in the same grade are recruited to meet ethical considerations of equal access to students from the same classroom. Children and their families are excluded if they are not interested in participating in the study, they move out of the district; they no longer have an eligible child living in their home, or do not comply with the completion of all survey instruments and anthropometric measurements. Families living within the Firebaugh-Las Deltas Unified School District (FLDUSD) are considered as the intervention group and those living in the Golden Plains Unified School District (GPUSD), which includes the town of San Joaquin, are considered participants in the comparison site.

Due to participants living in isolated communities with limited exposure to outsiders and the mixed immigration status of community members, developing trust between the research team and community members is important to allow for effective family recruitment and participation. A locally visible site location within the intervention community with Spanish-speaking staff is critical to project implementation. In addition, Spanish-speaking university researchers and students assist with participant recruitment. Several recruitment approaches are utilized including: (1) door-to-door recruitment (2) meet and greet events, (3) one-on-one/personal networks, (4) school events (back to school nights, child pick up drop off intervals), (5) Parent Teacher Meetings, (6) open market or *remate* informational booths, (7) community events (e.g., Day of the Dead, Red Ribbon Week), (8) religious organizations, and 9) community health fairs. In order to improve participation, food and raffles are offered. A master information sheet of all recruited families is used to track eligible, ineligible, refusal and dropout families. In order to track the completion of required activities for each family, an activity-tracking log records the progress. Each family must complete baseline surveys and anthropometric measures in order to join the program intervention activities in both sites.

Upon approval by UCD’s Institutional Review Board (IRB) of all data collection procedures and instruments, informed consent is obtained from legal guardians of eligible children, including fathers, mothers and/or grandparents. Informed consent is obtained through two primary methods: (1) face to face verbal review of all documents at the study field office or at participants’ homes, or (2) group presentations at the field office in the intervention site or at community events in the comparison site. A presentation is used for both individuals and groups in order to accommodate low literacy among participants and to facilitate better understanding of the research and assist in the consent process.

### Study instruments

The primary outcome of the study is the rate of BMI growth. We hypothesize that in the intervention arm, the rate of BMI growth will be less than the comparison community after three years of the intervention. We assess BMI, abdominal circumference and skinfold thickness in eligible children in both communities every six months. Anthropometric measurements are conducted in accordance with methods defined in the Anthropometric Standardization Reference Manual [16] and by a medical team consisting of trained staff and student surveyors from UCD, and trained local staff. Staff training consists of at least one, 3-hour anthropometric training and an observation session in which the trainees team up with experienced surveyors to gain hands-on experience. A digital scale by Seca (model 874) is used to weigh participants to the nearest 0.1 kg. A stadiometer (Seca 213) is used to measure height in centimeters (cm) to the nearest 0.1 cm. Abdominal circumference is obtained by using a QuickMedical QM2000 body circumference measuring tape. Finally, skinfold thickness of the triceps and subscapular regions are measured using Lange calipers (Beta Technology, Santa Cruz, CA).

Training and standardization manuals are developed and used in order to ensure precision and standardization of data. All the surveyors involved in measurement are standardized in order to assure accuracy and validity of measurements. The technical error of measurement (TEM) is used to calculate inter-observer and intra-observer errors [17]. For the inter-observer error, the surveyors compare their results to the lead anthropometrist. Standardization occurs before each anthropometric data collection period.

All measurements are conducted in duplicate. If the two measurements differ more than a certain pre-defined level, a third measurement is taken. At least two surveyors are involved in conducting anthropometric measurements. Steps are taken in order to protect the participants’ privacy and to minimize discomfort. In group settings, such as at the community center or at health fairs, anthropometric measurements are conducted in tents and participants are provided with disposable hospital gowns, and data are entered directly in the computer database. When measurements are conducted in participants’ homes, data are recorded on paper and are entered into a computer after leaving the home.

In order to fully understand underlying factors that may impact behavioral choice in these families, five surveys are administered at baseline and within defined intervals throughout the study (Table 2). Each participating family is required to complete baseline surveys as well as the anthropometric measurements to start the intervention. The surveys include: (1) United States Department of Agriculture Household Food Security tool (USDA-FS 2); (2) the Food Frequency Questionnaire (FFQ); (3) the Household Baseline Survey; (4) the Brief Acculturation Rating Scale for Mexican Americans (ARSMA); and (5) the Physical Activity Reporting Form (PARF).

**Table 2:** Variables, measures and instruments

**Survey measure**	**Data level**	**Instrument**		**Measured construct**	**Time Interval**	**Setting**
Anthropometric measurements	Individual	Weight	Digital scale	Anthropometric measures	6 months	School, health fair, NSFS office, participants’ homes
		Height	Stadiometer			
		Abdominal circumference	Body circumference measuring tape			
		Triceps skinfold thickness	Calipers			
		Subscapular skinfold thickness	Calipers			
Medical history	Individual Family	Medical History Survey		Family and personal medical history	1 year	Health fair, NSFS office, participants’ homes, phone-interview
Food security	Family	USDA Household Food Security Tool (FS)		Food Security	6 months	Health fair, NSFS office, participants’ homes
						Phone-interview
Nutrition	Individual	Food Frequency Questionnaire (FFQ)		Child eating patterns and feeding practices	6 months	Health fair, NSFS office, participants’ homes
						Phone-interview
Acculturation	Family	Brief Acculturation Rating Scale for Mexican Americans (ARSMA)		Acculturation	Baseline	Health fair, NSFS office, participants’ homes
						Phone-interview
Physical activity	Individual	Polar Accelerometer		Physical activity	1 year	School, health fair, NSFS office, participants’ homes
	Class	Class Physical Education Reporting Form		Physical activity	Weekly	Elementary and pre-school sites
Household baseline survey	Household	NHANES		Food purchasing patterns	1 year	Health fair, NSFS office, participants’ homes
		CPS		Demographic Information		
		NLSY		Income		
		ECLS-K		Expenditure		
		Local level WIC		Assets		

The USDA-FS includes 18 items. Food security is defined as 0–2 affirmative responses; low food security as 3–7 affirmative responses; and very low food security as 8 or more affirmative responses to the questionnaire [18]. This tool is used to monitor food security in the U.S. and is validated for use in a Mexican-origin population in California [19-21]. In these validation studies, food insecurity is correlated with lower household supplies of fruits and vegetables [22], which, in turn, is associated with lower intakes of these foods in preschool children [23]. Administration of the tool occurs using in-person and telephone interviews by trained students and staff surveyors. The in-person interviews are conducted at large gatherings, the field office and home-visits. Stations are set up with screens to provide privacy and assure confidentiality for all the participants.

We capture child eating patterns and feeding practices with the Food Frequency Questionnaire (FFQ) constructed of 30 questions and accompanied pictures. The tool includes 26 items related to the frequency of consuming specific foods over the past 30 days measured on a Likert scale. We selected 26 items based on studies previously validated in a binational study of Mexican-origin families [24]. In that study, an Americanized food pattern including frequent consumption of hot dogs, hamburgers, fried chicken, pizza, instant soup, instant cereal, and American cheese was significantly correlated with BMI-z-score in Mexican-origin preschoolers (r = +0.21, P < 0.0008) and demonstrated good internal reliability (Cronbach’s alpha =0.70). Four items are included in this scale to examine the structure of meals and snacks and parental role modeling at mealtimes, adapted from the Hughes Caregiver scale [25]. The development of this pictorial version of the FFQ includes a pilot testing with seven *promotores* from the intervention community. Only one FFQ is collected from each family regardless of the number of eligible children. Where more than one child is eligible, the parent is asked to respond for either a child between 4 and 5 years of age and who is not in kindergarten. If a child does not fall within these age categories, the response is based on the child closest in age to that window.

We use the Household Survey (HHS), which is designed and conducted to obtain information on food purchasing patterns, demographic information, income, expenditure, assets, and neighborhood characteristics of participating families. The household survey is based on previously tested and administered questionnaires from national and local levels [26-30]. Team members reviewed the surveys, compiled and edited questions that are relevant to the NSFS research objectives and study population. The survey was translated to Spanish and all questions were piloted with the intervention and comparison communities in order to ensure level of understanding of the questionnaire and elimination of questions as needed. An HHS manual was created to train surveyors. Surveyors completed a training course to learn about surveying techniques, data integrity, and the household survey instrument. After the initial training, new surveyors observe a survey being conducted by an experienced surveyor before conducting a survey. An experienced member also observes new surveyors during their initial interviews. Household surveys are collected by in-person interviews. Surveyors administer the surveys using netbooks and capture data electronically. The average time to administer the survey is 45 minutes for a seasoned surveyor, but varies depending on family size and situation. Surveyors complete a survey log to indicate surveyor, duration of the survey, language used to complete the survey and any annotation or information that might need to be clarified during the cleaning stage of the data.

Participating families in both communities answer the Brief Acculturation Rating Scale for Mexican Americans (ARSMA) [31]. The ARSMA consists of two subscales, an Anglo Orientation Subscale and a Mexican Orientation subscale. Within this population the ARSMA scale is used extensively with good internal reliability and strong construct validity and is only administered at baseline [31].

To track school-based physical education, pre-school and K-2^nd^ grade teachers in the intervention group are asked to record their daily delivery of formal (i.e. teacher-led, structured) physical activity (excluding recess) using a weekly form developed and tested by the researcher and teachers. K-2^nd^ grade teachers in the comparison district are asked to not change their regular physical activity levels and to record formal classroom physical activity sessions (excluding recess) on the same reporting form as the treatment teachers. Both groups are asked to identify each day of the week and minutes each day that children are engaged in formal or in-class physical activities. For each physical activity session, teachers record the specific activity performed by the students and the level of exercise noted as Low, Medium, or High. These data are turned into their supervisors and collected by program staff.

In addition to the survey tools, we use other methods of data collection. In order to measure actual household food purchase/consumption data, we collect retail store purchasing data. Scanners are installed in the Firebaugh Supermarket and families are given a loyalty card to use at the store during every transaction. Data for every item purchased is automatically recorded through the scanning software. These scanner data will be collected during the intervention and follow-up years from participants in the intervention community of Firebaugh and automatically sent and stored in a central server. In addition, families are instructed to collect and turn in food receipts from stores other than Firebaugh Supermarket. In the comparison group, all food receipts will be collected during the monthly nutrition workshops. These data will be transferred to a central database and linked to the household data set.

**Figure 2:**
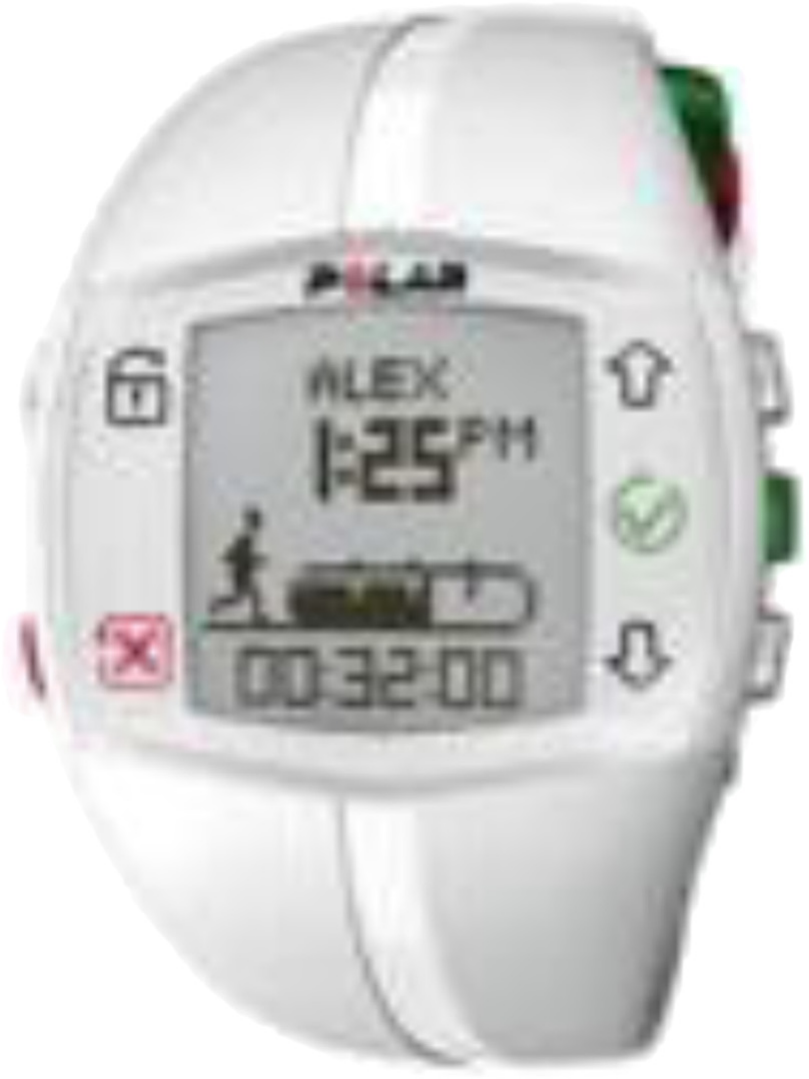
Polar™ Active accelerometer.

Physical activity is assessed in a subset of eligible children enrolled in the study once per year using a PolarActive™ unidirectional accelerometer (Figure 2). This device provides continuous 24-hour measurement of total energy expenditure (kcal), number of steps, sleep duration, and time spent in each of five intensity zones (very easy, easy, moderate, vigorous, and vigorous+). The development and validation of this device have previously been described [32]. Briefly, physical activity measurement with this device has shown good correlation (R = 0.93) with oxygen consumption measurement in children [32].

The accelerometer protocol was developed to target participants aged 5–7 y. Each eligible child uses the device which is worn on the wrist for seven consecutive days, allowing for the collection of five complete days of data inclusive of at least one weekend day. Children and their parents are verbally instructed to not remove the accelerometer during the period of use, including during water-based activities (e.g., bathing, swimming) and while sleeping. Parents are provided a diary to note any removals, including the time, duration and reason for removal. As part of the protocol development, a qualitative pilot was conducted with children in a rural community similar both culturally and socioeconomically to the target communities in this study, to test device acceptability and protocol adherence.

Evaluation of the community arts projects in both the intervention and comparison communities relies on a qualitative methodology. To assess the impact of the community art projects, focus groups are conducted. Kieffer et. al (2005) suggest that focus groups be used to help researchers build a deeper understanding of the community and the impact of the intervention on the community [33]. In attempts to quantify these impacts and change in the level of civic engagement, the focus group data will be used to develop a community engagement survey that will be implemented in year 2, 3, and 4 of the intervention. The instrument will be piloted in a rural community that will serve as comparison for the validation of the instrument prior to implementation.

#### Data collection methods

This project applies several data collection formats, including data collection on paper, directly into netbooks and by accelerometer. All paper-collected data is entered into spreadsheets and double-checked for data entry quality through pre-defined steps. We use netbooks (Gateway® LT2000) to collect survey data. All the surveys are designed into the LimeSurvey™ (Version 1.91) on netbooks. Data from netbooks is backed up on regular basis and eventually stored in a central server. Data collected by accelerometer are uploaded to the *polargofit.*com web service and a report of each child’s activity per 24-hour period is produced in Microsoft Excel format.

### Intervention

The NSFS project consists of four major component interventions in the areas of nutrition, physical activity, economic and art-community engagement to induce behavioral change in our target population in order to achieve obesity prevention (Table 3). Each component is explained in detail below.

**Table 3:** Description of intervention components

**Behavior area**	**Focus**	**Intervention**	**Target**	**Frequency**	**Intensity**
Nutrition	Family nights	Educational workshop focusing on nutrition and health messages	Any adult member of each eligible family	Monthly	One hour
	School curriculum	Nutrition education by UCCE in elementary school and preschool	Eligible child		
Physical Activity	School curriculum	SPARK physical activity program in elementary school and preschool	Eligible child	4 days/week	20 minutes
Economic	Voucher	Each family with one eligible child receives a $25 voucher/month that can be used towards the purchase of fruits and vegetables	Family of eligible child	Monthly	$25/month/family
Art community engagement	Community events	Art projects in community or messaging healthy behaviors	Community	NA	NA

#### Nutrition

The nutrition intervention consists of two components a) family nights and b) a school-based program. Family nights are led by a local health educator and are based on a series of focus groups to understand community perceptions of childhood obesity including barriers, opportunities, interpretation and prioritization of key prevention messages recommended by the American Academy of Pediatrics [34]. The nutrition team consists of University of California, Cooperative Extension (UCCE) nutrition staff, advisors and specialists; UCD faculty and graduate students; and local staff based in Firebaugh. Based on focus group data and findings from other studies, the team develops a three-year plan for family nights, including key messages, recommendations on logistical issues (space, length of classes), and a plan for evaluation. In this study, cultural adaptation begins with nutrition curricula and approaches that work well in the general low-income audience served by UCCE. The local health educator who lives in the community is trained in basic nutrition, safe food handling, adult education and learner-centered delivery techniques and meets with the nutrition specialist each month to modify messages and activities. Adapted lessons are piloted with one group of participants to incorporate group feedback before delivering the lesson to the rest of the families. Each lesson is approximately one hour in duration and includes facilitated discussion, a hands-on activity (such as label reading or meal planning), and a food demonstration and tasting of a recipe containing fruits and/or vegetables. Families are required to attend at least five of the ten classes offered each year (15 minimum over three years) to remain in the program. Any adult member of each eligible family can participate in the family nights to receive credit for attendance.

In coordination with UCCE Fresno County, school-based nutrition education is delivered to children in the classroom. Staff from the UCCE, CalFresh and Expanded Food and Nutrition Education Program (EFNEP) are involved in program support. Age-appropriate UCCE curricula include “Happy Healthy Me”, “Reading Across MyPyramid” and “Farm to Fork”. A UC CalFresh program representative provides the lesson plans, foods for tasting activities and other materials to the classroom teachers, along with training on how to deliver the lessons. The classroom teacher determines the actual number of lessons taught each year. Routine program evaluation provides records of teacher perception of student improvement in nutrition skills and behaviors and the number of lesson delivered.

#### Physical activity

The physical education curriculum used for students in the intervention arm is based on the Sport Play and Active Recreation for Kids (SPARK) curriculum. Materials include the Early Childhood (ages 3–5) curriculum binder and the K-2 curriculum binder [35]. Each curricular component is presented in scope and sequence via daily lesson plans that are aligned to the National Association for Sport and Physical Education (NASPE) Standards. The SPARK K-2 curriculum also contains academic integration tips (with a special emphasis on literacy), social skills themes by grade level, and challenging extensions for each activity [36]. The SPARK curriculum was chosen for the school physical activity intervention based on its successful use in several studies [37].

In grades K-2, implementation of the physical activity curriculum is carried out by a local teacher trained in SPARK and employed by the intervention school district. During the intervention, the SPARK trained teacher works with 19 K-2^nd^ grade teachers and approximately 300 children each week, delivering 20–30 minute exercise sessions to each class. All 19 K-2 grade teachers receive SPARK curriculum binders and CDs and share the use of SPARK equipment, housed in a central storage area on the school campus. In addition to the physical activity sessions delivered by the teacher, all K-2^nd^ grade teachers integrate SPARK activities into their class lessons so that the children receive an average of 66 minutes of physical education each week.

For intervention site preschools, a program research staff member trained in SPARK Early Childhood assists the preschool teachers in integrating the curriculum into their lesson plans. Twelve teachers from eight pre-schools sites receive Early Childhood SPARK curriculum and equipment and dedicate an average of 71 minutes each week to the activities.

#### Economic

The economic intervention consists of providing participating families in Firebaugh with monthly fruit and vegetable vouchers worth $25/month. Participating families receive the monthly payments throughout the three-year intervention period. This approach provides incentives to families to purchase more nutritious and healthier foods. Vouchers are allocated per family regardless of the number of eligible children, family size or composition. A “family” for voucher purposes is described as an eligible child and a guardian, and the members directly related to them who consume food using the same resources. The list of fruits and vegetables that families can purchase using their vouchers matches the list of fruits and vegetables used by the WIC program. Unused voucher money expires at the end of the month and the card is automatically reloaded. Vouchers can only be used at the Firebaugh Supermarket and market staff are responsible for ensuring that funds are spent on approved purchases. Families must attend nutrition classes or check-in with the NSFS staff on a monthly basis to continue receiving funds. If a family does not check-in during a given month, their card is frozen until they regain contact with program staff. This ensures that the cards are still within the participant’s possession and have not been lost or stolen. Additionally, NSFS staff collect food purchase receipts from participants on a monthly basis and transfer these receipts to the UCD team for processing.

#### Community art

Community art tools and strategies, including murals, are used to engage and involve community members. A summer mural project in the comparison community of San Joaquin is carried out through collaboration between UCD faculty, staff, undergraduate students, as well as members of the community including local high school students. The UCD team recruits local community members to participate in mural development activities, including mural content meetings. Once the content is finalized, the team spends approximately two weeks in the community painting the mural. Through this community-based project, members of the UCD team engage local high school students in discussions about their community’s culture and history and about their educational aspirations.

A corresponding series of community-based art interventions also occur within the intervention community of Firebaugh. These interventions include a series of limited edition screenprints that provide a visual representation of the proposed interventions.

### Statistical analysis

#### Sample size calculation

During the baseline phase of the study, all eligible children and their families in both Firebaugh and San Joaquin are approached. Based on an initial estimation of the total number of children from previous years, we anticipated that our sample would contain approximately 400 children in each group. This number of subjects is sufficient to detect an effect difference between groups in the mean change in standardized BMI (z-scores) of as small as 0.20 standard deviations with 80% power. In a previous study with Latino school children, a change of that magnitude was achieved over a two-year period.

Our sample constructs a multi-cluster formation of students, classrooms, grades, schools and cities. Because we are not assigning multiple cities to each group, we have to assume that the matching of cities will take care of any clustering problem at the city level. However, accounting for clustering is a problem in sample size calculation because there is really no way of estimating in advance the within-cluster correlation (r) with respect to any of the outcome variables. To our knowledge this information is not available from other studies. If we assume that r = 0.01 among school grades in schools and there are an average of 143 students per grade then based on the design effect formula (DE = 1 + (n-1)*r), the detectable effect difference in primary outcome will be 1.56 * 0.20 standard deviation with 80% power in this study. Based on the school’s estimation there will be less than a 5% mobilization rate within the targeted schools. All new students who fall in our sampled cohort will be eligible for the study.

## Discussion

Careful consideration and strategies are required to maximize recruitment and retention of this hard to reach population of both the intervention and comparison communities. An important consideration is that farm work employment peaks during the months of April to October when many family members work long hours in the field. Strict adherence to using *promotores*, local staff, and/or field researchers to contact participants is required to contact participants to conduct informed consent, surveys and medical measurement. Further, relying on standard appointments for participants does not guarantee they show up at appointment time. It is therefore often required that program staff make visits to the families’ homes to conduct recruitment and/or data collection procedures. In addition, program staff works with the school nurse to schedule anthropometric measurements of children during the school day.

Recruitment and retention of participants for the comparison community of San Joaquin also presents a unique set of challenges. For example, the comparison site lacks office space that would provide visibility to the project in that community. Because of funding limitations, project staff use space available from local organizations such as churches and the community center. This may compromise the stability of staff access to the community at large in the comparison community. Also, since there is no active nutrition intervention in San Joaquin, project staff anticipated more problems with recruitment and retention. Therefore, focus groups were used to develop a series of workshops that focus on family well-being and ways in which parents can support their children’s education. These will be offered monthly to all community members. Workshop attendees invite other community members who are eligible to participate in the study. This strategy increases the community members’ interest in NSFS and the enrollment of participants.

In the implementation of the nutrition education component, careful selection of adequate space is critical to establish an environment where adults can relax and learn. However, a main challenge is securing a suitable site in the community for family night, i.e., a safe, quiet, adequate space for childcare, and a convenient location. For example, a potential site (a closed school site) seemed to meet all criteria but was later found to harbor asbestos. Securing access to a second site, a classroom in the local community college, required more than six months of negotiation between UCD and the college to formalize liability management.

Another critical factor for success of nutrition education is cultural adaptation and tailoring of nutrition messages. Interventions delivering culturally-adapted, family-centered nutrition education to Latino families with preschool children have reported improvements in BMI status of the children [4,5]. To make the content culturally relevant and engage participants in this study, the local educator must use open-ended, problem-solving questions in small group discussions (10–12 parents). Food demonstrations of recipes, a monthly raffle, and free childcare were other aspects of the Family Nights that served as a cultural draw.

A number of administrative issues occurred during the initial implementation of the fruit and vegetable vouchers. The voucher payments are distributed via automatically reloading US Bank debit-style cards. During the initial month of distribution, US Bank switched the reload date from the first of the month to the 25^th^ of the month without previous notification. Furthermore, there is a delay in processing transactions between the retails store and US Bank. For these reasons, card use has been restricted between the 20^th^ and 28^th^ of each month. Participants chose to have restrictions on use rather than receiving a larger allotment of funds at one time because it would be too cumbersome to track larger balances. The balance on each card is not reported at the time of use and balance check information through US Bank is only available in English. Due to these factors, Spanish-speaking participants must individually keep track of remaining funds or call the field office for assistance.

The retail environment provides another set of challenges toward implementation. Retail storeowners and employees are required to be trained on voucher use and protocol. The economic voucher may be used with other forms of payment and employees are not used to these types of complicated transactions. Furthermore, participants are not able to combine the WIC fruit and vegetable voucher with the economic voucher during the first four months of intervention. Store employees process WIC payments in a way that does not allow them to be combined with other forms of payment. A large number of our participants also participate in WIC and are therefore inconvenienced by this issue.

Collection of scanner data also imposes complications. The retail store in the comparison community severed our relationship just prior to implementation. They were not going to receive a financial incentive to allow scanner data collection and decided it was not in their interest to participate. Scanner data collection has been ongoing in the intervention community but needs to be translated by a computer programmer for analysis. Furthermore, scanner systems are extremely technical and need to be constantly monitored by specialize staff.

The implementation of the physical activity intervention was helped significantly by the decision of superintendent to support physical activity at the intervention site by hiring a local teacher to teach SPARK physical activity to K-2^nd^ graders each week. However, a challenge for the physical activity intervention occurred when the team realized that lead teachers attending one SPARK training were not allowed by the SPARK Program to return to the schools to train other teachers. SPARK requires that 50% of the teachers attend the SPARK training or SPARK trainers come to the district to train the teachers. Unfortunately, both options required funding levels beyond the budget. However, it was learned that the SPARK curriculum is available for direct purchase for teachers to use in their classrooms without formal training, as the binders are very clear and easy to follow. The research team decided to buy the SPARK curriculum and equipment for the teachers and although the teachers received no formal training, the K-2^nd^ grade teachers were able to watch the local SPARK trained teacher instruct the lessons and they could then duplicate this lessons in their classrooms. The physical activities at the pre-school levels were very basic and pre-school teachers easily integrated the activities into their daily activities with minimal coaching from the research team.

In sum, the unique environmental condition of the study that is rural and isolated locations with limited infrastructure capacity, the distance of the university research team from the local community, combined with limited resources and unstable population with limited exposure to researcher expectations necessitated adaptation reassessment of recruitment strategies and data collection methods by the research team.

## Trial status

At the time of submission, the study is continuing the recruitment of families in both intervention and comparison arms and at the same time the intervention has started in the intervention community.

## Abbreviations

ARSMA: Brief acculturation rating scale for Mexican Americans; BMI: Body mass index; CAC: Community advisory council; CBPR: Community-based participatory research; CDC: Centers for disease control and prevention; cm: Centimeter; FFQ: Food frequency questionnaire; FLDUSD: Firebaugh-Las Deltas Unified School District; GPUSD: Golden Plains Unified School District; HBM: Health belief model; HHS: House hold survey; IRB: Institutional Review Board; kcal: Kilocalorie; kg: Kilogram; mm: Millimeter; MVPA: Moderate-to-vigorous physical activity; NIFA: National Institute of Food and Agriculture; NSNF: Niños Sanos, Familia Sana; NHANES: National Health and Nutrition Examination Survey; (PARF): Physical Activity Reporting Form; PE: Physical education; RAMP: Reading across My Pyramid; SCT: Social Cognitive Theory; SPARK: Sport play and active recreation for kids; TEM: Technical error of measurement; UCCE: University of California, Cooperative Extension; UCD: University of California, Davis; US: United States; USDA: United States Department of Agriculture; USDA-FS: United States Department of Agriculture Food Security Tool; WIC: Women, Infants and Children.

## Competing interests

The authors declare that they have no competing interests.

## Authors’ contributions

AdT integrated and developed the protocol design, coordinated and drafted the manuscript, and provided overall oversight and editing for the final draft. BS oversees the collection of anthropometric measurements, data management and drafted the manuscript. RDG participated in the design of the economic portion of the study, the statistical analysis, data analysis, the development of economic models, and in the econometric approaches to estimate the models. LK authored the section on the design and evaluation of the nutrition education component of the intervention, including selection of the instruments to measure food security, child feeding practices, and frequency of consuming specific foods. YGF authored the methodology of the comparison community. CFJ contributed to the formulation of the community art engagement methodology. SES developed and wrote about the methodology for collecting physical activity data on children using accelerometers, and edited the manuscript. US participated in the design and implementation of the study. LW coordinates the implementation of the school-based physical activity component. All authors read and approved the final manuscript.

## Pre-publication history

The pre-publication history for this paper can be accessed here:

http://www.biomedcentral.com/1471-2458/13/1033/prepub
